# Neoantigen Quantity and Quality in Relation to Pancreatic Cancer Survival

**DOI:** 10.3389/fmed.2021.751110

**Published:** 2022-02-09

**Authors:** Iris J. M. Levink, Lodewijk A. A. Brosens, Sander S. Rensen, Merel R. Aberle, Steven S. W. Olde Damink, Djuna L. Cahen, Sonja I. Buschow, Gwenny M. Fuhler, Maikel P. Peppelenbosch, Marco J. Bruno

**Affiliations:** ^1^Department of Gastroenterology and Hepatology, Erasmus MC University Medical Center, Rotterdam, Netherlands; ^2^Department of Pathology, University Medical Center Utrecht, Utrecht University, Utrecht, Netherlands; ^3^Department of Surgery, NUTRIM, School of Nutrition and Translational Research in Metabolism, Maastricht University, Maastricht, Netherlands; ^4^Department of General, Visceral and Transplant Surgery, University Hospital Aachen, Aachen, Germany

**Keywords:** cancer immunity, pancreatic cancer, neoantigen and shared-antigen vaccine, mutation–genetics, chromosomal instability disorders

## Abstract

**Introduction:**

Factors underlying antitumor immunity in pancreatic cancer (PC) are poorly understood. We hypothesized that not neoantigen quantity, but quality, is related to immune cell infiltration and survival.

**Methodology:**

We performed genomic and transcriptomic profiling of paired normal, tumor tissue of 13 patients with PC with distinct survival times. Additionally, neoantigens prediction and immunological profiling were performed.

**Results:**

The proportion of neoantigens with a low similarity-to-self score was higher in short-term survivors (*p* < 0.0001), while mutational load and burden, similarity-to-known-pathogens, and immunogenicity of neoantigens were not associated with immune cell infiltration or survival.

**Discussion:**

No tumor mutational load or neoantigen quantity, but low similarity-to-self score, was associated with immune cell infiltration and survival.

## Introduction

The efficacy of inducing an antitumor immune response depends on the major histocompatibility complex (MHC) presentation of tumor-specific antigens, as well as on the ability of the immune system to recognize these antigens as “non-self” and initiate a durable tumor-specific response (1). In highly mutated cancers, tumor neoantigen quantity is related to CD8^+^ T-cell infiltration. For pancreatic cancer (PC), high CD8^+^ T-cell infiltration has been related to improved survival. While PC lesions have a relatively low mutation rate, PC survival has been reported to be not related to the quantity of MHC-binding neoantigens *per se* (2). Nevertheless, a high number of neoantigens combined with high CD8^+^ T-cell infiltration correlate with unusual long-term survival (median 6 years) (2). This long-term survival has also been related to higher neoantigen quality as defined by the similarity of an antigen to known disease-derived peptides (2).

As PC survival over 2 years is rare, it is important to know whether differences in neoantigens and immune infiltrate are also relevant on a shorter time scale. Additionally, a model is needed to characterize the tumor and predict response to immunotherapeutic agents (preferably prior to surgery). Pancreatic organoids may provide such a model, as these can be established from a histologic biopsy obtained with fine-needle biopsy early in the diagnostic process of PC (3, 4).

Hence, the aims of this study are to investigate neoantigen quantity and quality in relation to immune cell infiltration and PC survival time, as well as to explore the potential of organoids as a model for neoantigen prediction.

## Methods

### Study Design

This study involved patients diagnosed with PC who underwent resection for PC from two University Medical Centers (Utrecht and Maastricht), who underwent pancreatic surgery between 2007 and 2015. The cohort was divided into long-term survivors (survival ≥ 14 months after surgery) and short-term survivors (survival ≤ 11 months after surgery).

Tumor and normal adjacent tissue were obtained during pancreatic resection and frozen. For two patients, organoids were cultured from tumor tissue. Clinical data were extracted from patient records.

### Organoid Culture

Pancreatic tumor organoids were established according to previously described protocols at the Department of Surgery of Maastricht University Medical Center (5). Upon arrival after resection, the tumor tissue was minced, washed with Advanced DMEM/F12 (Gibco, Cat. No. 12634-010), supplemented with PenStrep, Glutamax, and HEPES (AdvDF+++), and digested with collagenase II (5 mg/ml, Gibco, Cat. No. 17101-01) in AdvDF+++, supplemented with a 50% (v/v) Wnt3a conditioned medium (CM) (6) and a 10-μM Rho Kinase inhibitor (Y-27632) on an orbital shaker at 37°C for 1–2 h. The digested tissue suspension was further digested with TrypLE (Gibco, Cat. No. 12605-010), supplemented with a 10-μM Rho Kinase inhibitor at 37°C. TrypLE digestion was stopped by adding ice-cold AdvDF+++, followed by 5-min centrifugation at 350 x g at 4°C. Subsequently, the pellet was resuspended in ice-cold basement membrane extract (BME; Geltrex LDEV-Free Reduced Growth Factor Basement Membrane Matrix, Gibco, Cat. No. 1413202), and droplets of the suspension were allowed to solidify in 24-well culture plates (Eppendorf). When the droplets were solidified, 500 μl of either organoid-medium “a” or medium “b” (5) was added to each well. For Patient #13, this resulted in the establishment of two independent organoid cultures (in two mediums: “a” and “b”) from one individual tumor biopsy; for Patient #11, one organoid culture (medium “b”) was established. The plate was transferred to a humidified 37°C/5% CO_2_ incubator, and the medium was changed every 2–3 days. The organoids were passaged every 7–10 days. Organoids were collected in AdvDF+++ and mechanically sheared through narrowed glass Pasteur pipettes. Following centrifugation at 350 x g (5 min, 4°C), organoid fragments were resuspended in ice-cold BME and plated as described above.

### DNA and RNA Isolation

DNA and RNA were co-isolated using the AllPrep DNA/RNA Tissue Kit (Qiagen, Germantown, MD). A total of ≥ 100 ng of co-extracted DNA and RNA from a single sample was further utilized. From patient #13, DNA and RNA extracted from the two independent organoid cultures grown in different culture media were pooled to ensure maximal representation of the parent tumor.

### Genomic Analysis

Whole-exome sequencing of DNA from the tumor, organoids, and normal tissue was performed using Illumina NovaSeq instrumentation with a read length of 2 x 150 bp and standard configuration ± 120 X covering > 20,000 genes (> 1,400 cancer-related genes; Personalis). To align reads with the hs37d5 reference genomes and produce a set of single nucleotide variant calls (SNVs), BWA-MEM (version 0.7.12) was used in combination with Mutect (from Appistry Cancer Analysis Package 2014.1-13-g6b71cb4), Vardict (Java Vardict 1.4.3). Vardict is also used to call small somatic insertions or deletions of <50 bp. Subsequently, the set of SNVs and indels from tumor tissue and organoids was corrected by the following quality parameters: alignment metrics (e.g., coverage and read quality), positional features (e.g., proximity to a gap region), presence in normal tissue, an allelic fraction (> 0.05%), and the frequency in the general population.

HLA typing is performed using the HLAssign tool (7). This tool used the whole exome sequencing data to perform accurate genotyping of HLA Classes I and II loci.

### Neoantigen Perdiction

For antigen prediction, the Neoantigen ID pipeline was used (Personalis, Menlo Park, CA). All SNVs and indels were considered as potential neoantigens. For binding prediction, peptides with a length of 8–11 amino acids were considered as binders for MHC Class I and peptides with a length of 14–21 amino acids for MHC Class II. MHC-binding prediction was performed on the complete set of peptides using NetMHC (v 4.0) and NetMHCpan (v 3.0) for MHC Class I peptides (HLA-rank ≤ 2) and NetMHCIIPan (v 3.2) for Class II peptides (HLA-rank ≤ 10) (8–10). Neoantigen quantity was defined as all HLA-peptide combinations with a predicted binding score of HLA-rank ≤ 2 for MHCI and HLA-rank ≤ 10 for MHCII. In parallel, both gene and variant level expression data are described, which can be utilized as a powerful filtering tool for potential candidates. Additionally, immunogenicity score was calculated based on the ability of a given peptide MHC-complex to be recognized by T-cells, which was based on (1) position of a presented peptide P4–P6 for Class I; (2) The type of the amino acid (i.e., aromatic vs. non-aromatic, acidic vs. basic, and charged vs. non-charged) (11). Increasingly positive scores indicate higher immunogenicity. Furthermore, predicted neoantigens were compared to neoantigens in the normal tissue or to the full epitope database from IEDB (http://www.iedb.org), excluding human epitopes. The higher the score, the more similar the neoantigen is to either self-antigens (similarity-to-self score) or antigens from known pathogens (similarity-to-known score).

### Transcriptional Analysis

RNA sequencing (RNAseq) of tumor tissue and organoids was also performed using Illumina NovaSeq instrumentation (read length 2 x 150 bp) with standard configuration 25- to 50-M paired-end (50–100 M total) reads, covering > 20,000 genes (Personalis, Menlo Park, CA). According to the best practice workflows by the Broad Institute (https://gatk.broadinstitute.org), STAR alignment was used during post processing of RNAseq data. Post-processed BAMs were further processed such that map qualities have been reassigned, duplicates were removed (novosort), indels were realigned [Genome Analysis Toolkit v2.8.1 (GATK)]. Subsequently, the bases were recalibrated following GATK best practices Institute (https://gatk.broadinstitute.org). Aligned sequence data are then returned into BAM format according to the SAM specification (12). The STAR aligner subsequently generated raw counts and normalized expression values [Counts per Million mapped reads (CPM)], Fragments per Kilobase per Million mapped reads [FPKM], and transcripts per million [TPM] for genes in the given assay.

CIBERSORTx (13) was used on RNAseq data (transcripts per million). LM22 (14) was selected as a signature matrix file, which is designed and extensively validated on gene expression data and comprises of an algorithm, taking into account the expression of 547 genes related to the presence of 22 immune cells. The relative gene expression profile of these genes in 22 immune cells (and their differences) can be found in Supplemental Table 1 of Newman et al. (14). The absolute values were used for group comparisons.

To analyze the T-cell receptor repertoire MiXCR tool (15) was used, which enables comprehensive analysis of both TCRα and TCRβ chains [RNAseq; complementarity-determining regions 3 (CDR3)] of T-cells present in tumor samples and provides TCR clonality (Shannon entropy). Shannon entropy was calculated on the clonal abundance of all productive TCR sequences in the data set as described before (16) and normalized by dividing Shannon entropy by the logarithm of the number of unique productive TCR sequences in the data set. This normalized entropy value was then inverted (1—normalized entropy) to produce the clonality metric.

### Statistical Analysis

For survival analysis, the *p*-value was calculated using a log-rank. In case of comparison of investigated groups, a Mann–Whitney U test or ANOVA (for more than two groups) was performed, while, for comparisons of proportions, an X^2^ test or Fisher exact test (in case of low numbers) was used. For correlations, a Spearman correlation was conducted.

A (two-sided) *p*-value of <0.05 was considered significant. Data were analyzed with Statistical Package for the Social Sciences (SPSS), (SPSS Inc., Chicago, IL). Figures were created by GraphPad (GraphPad Prism version 9, GraphPad Software, La Jolla, CA).

## Results

**Figure 1:**
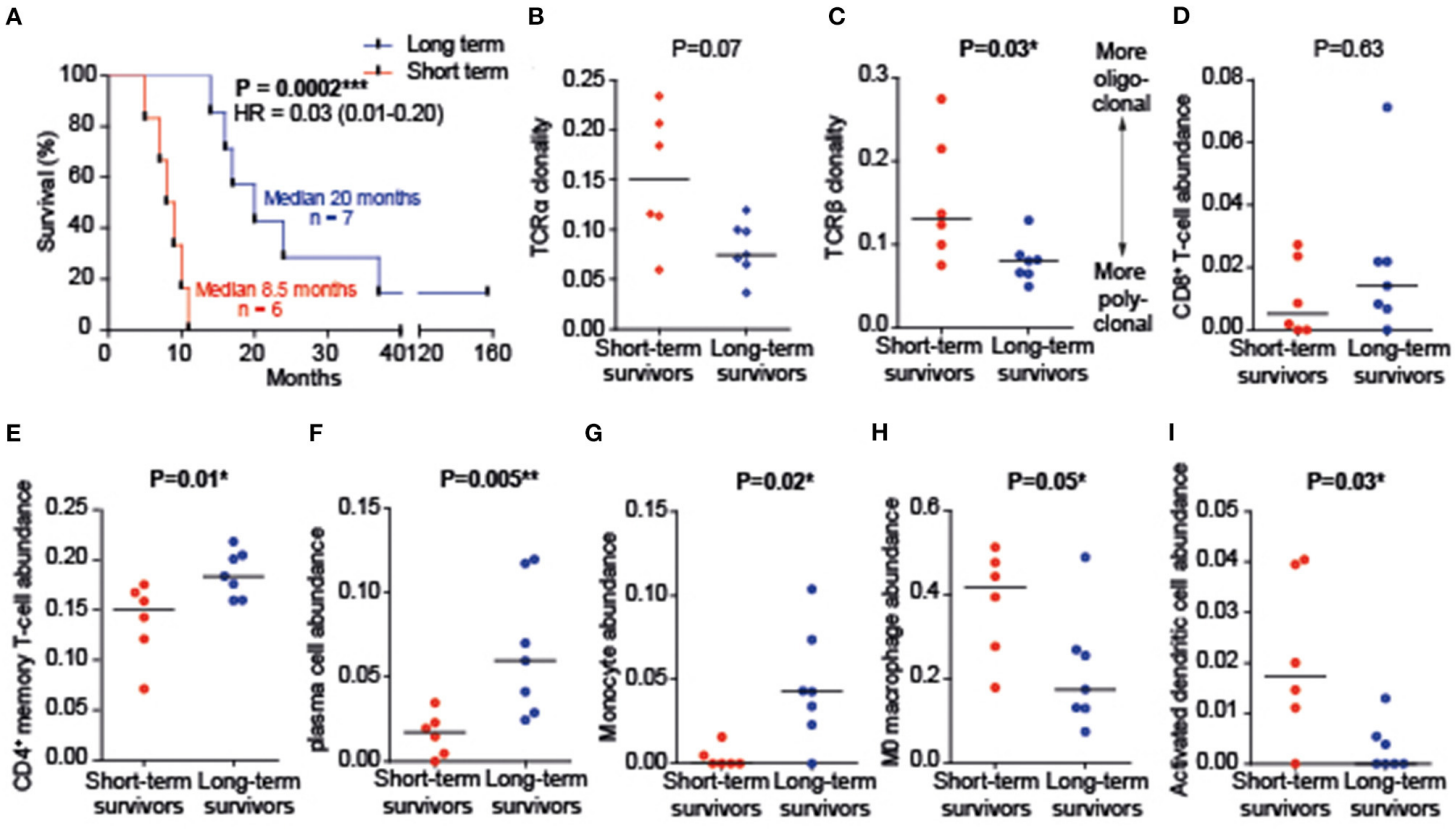
Immune cell infiltration. **(A)** Survival time of two survival groups. HR, hazards ratio. **(B,C)** T cell receptors (TCRs) of short-term survivors are more oligoclonal, while TCRs of long-term survivors are more polyclonal. TCR clonality was based upon Shannon's entropy. **(D–I)** Immune cell abundance as determined by CIBERSORTx (signature Matrix: LM22). An abundance of B-cells, regulatory T-cells, NK-cells, M1/M2 macrophages (ratio and absolute expression), mast cells, eosinophils, and neutrophils did not differ between groups and were, therefore, not shown. *P < 0.05, **P < 0.01, ***P < 0.001.

For this study, we included 13 patients with PC that had not received prior chemotherapy (Table 1). The cohort was divided into seven long-term survivors (median survival, 20 months) and six short-term survivors (median survival, 8.5 months; HR = 0.03; 95% CI, 0.01–0.2; *p* = 0.0002; Figure 1A). At the baseline, survival groups were similar regarding age, gender, and tumor stage (*p* < 0.05).

**Table 1:** Clinical characteristics.

**Patient**	**Survival group**	**Survival (months)**	**Age/gender**	**BMI**	**Tumor location**	**Resection margin**	**No. of positive lymph nodes**	**Tumor stage (AJCC 8th edition)**	**Adjuvantchemotherapy**
#1	LTS	157	40/F	22.2	Head	R0	1–3	IIB	Gemcitabine
#2	LTS	37	74/M	26.5	Head	R1	0	IA	Gemcitabine
#3	LTS	24	60/F	23.2	Head	R1	≥ 4	III	Gemcitabine
#4	LTS	19	74/M	21.9	Head	R0	1–3	IIB	Gemcitabine
#5	LTS	17	63/M	23.5	Head	R0	≥ 4	III	Gemcitabine
#6	LTS	16	57/M	23.1	Head	R0	1–3	IIB	Gemcitabine
#7	LTS	14	62/F	25.6	Head	R1	≥ 4	III	Gemcitabine
#8	STS	11	78/M	24.2	Head	R0	≥ 4	III	Gemcitabine
#9	STS	10	65/M	24.3	Head	R0	0	IB	Gemcitabine
#10	STS	9	70/F	27.1	Head	R0	0	IB	Gemcitabine
#11	STS	8	62/F	23.6	Tail	R1	≥ 4	III	Gemcitabine
#12	STS	7	29/F	20.4	Head	R1	≥ 4	III	Gemcitabine
#13	STS	5	78/M	28.4	Head	R1	≥ 4	III	No

*F, female; M, male; BMI, body mass index; LTS, long-term survivor; STS, short-term survivor*.

Deconvolution of tumor infiltration of 22 immune cell types revealed no differences in CD8^+^ T cell abundance between survival groups (Figure 1B). T cell receptors (TCRs) of long-term survivors, however, were more polyclonal (TCRα *p* = 0.07; TCRβ p = 0.03; Figures 1C,D). Tumor tissues of long-term survivors were rich in plasma cells (*p* = 0.005), CD4^+^ memory resting T cells (*p* = 0.01), and monocytes (*p* = 0.02), while naive (M0) macrophages and activated dendritic cells (DCs) were more abundant in tumor tissues from short-term survivors (Figures 1E–I).

**Figure 2:**
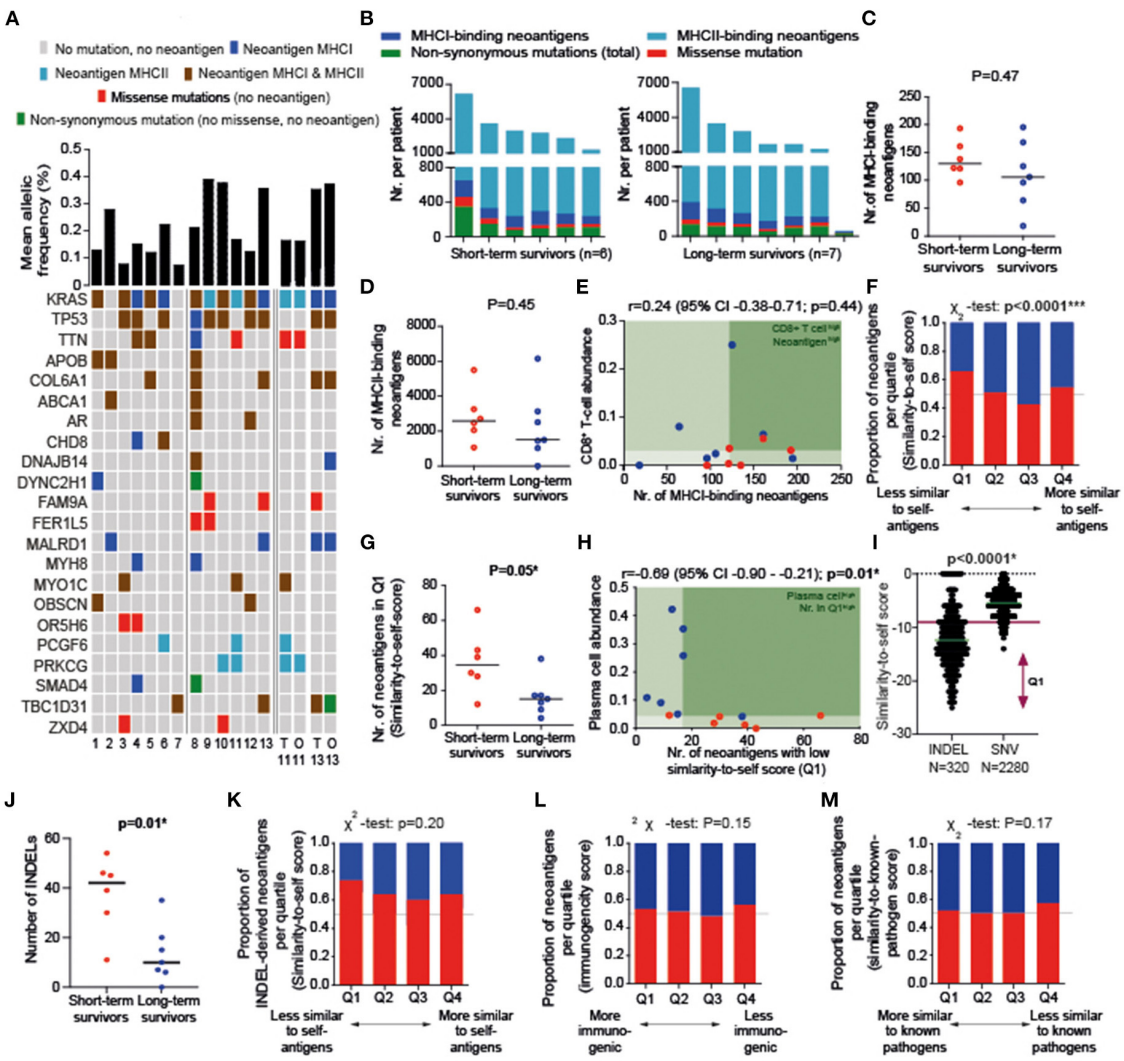
**(A)** An overview of mutations present in individual patients (Patient #1-13; ordered based on survival time from left to right) and whether mutation resulted in an MHCI- or MHCII-binding neoantigen. All mutations present in ≥ 2 patients are displayed. The majority of presented gene mutations overlap between tissue (T) and organoids (O) for the two patients from whom organoids were available (#11, #13). **(B)** Number of mutations and neoantigens per patient. **(C,D)** Quantity of MHCI- and MHCII-binding neoantigens did not differ between survival groups. **(E)** In contrast to previous literature, high neoantigen quantity in combination with high T-cell infiltration is not associated with long-term survival. **(F)** The majority of Q1-antigens (least similar-to-self antigens) were detected in short-term survivors (cut-offs: Q1 ≤ −9, Q2 −8 through −6, Q3 −5 through −4, Q4 ≥ −3). **(G)** The number of least-similar-to-self antigens (Q1) was higher in short-term than long-term survivors. **(H)** A low number of neoantigens with a low similarity-to-self score (Q1) is correlated with high plasma cell infiltration. **(I)** A low similarity-to-self score is associated with the presence of an INDEL. **(J)** A high number of INDELs is associated with short-term survival. **(K)** The majority of Q1 INDEL-derived neoantigens were detected in short-term survivors (cut-offs: Q1 ≤ −16, Q2 −15 through −13, Q3 −12 through −9, Q4 ≥ −8). **(L,M)** The number of high or low similarity-known pathogen [**(L)**; cut-offs: Q1 ≤ −23, Q2 −19 through −22, Q3 −16 through −18, Q4 ≤ −15] and immunogenicity scores [**(M)**; cut-offs: Q1 ≥ 0.1435, Q2 0.0185–0.1434, Q3 −0.1189–0.0185, Q4 ≤ 0.1188] do not differ between survival groups. *p*-values were calculated using Mann–Whitney U test **(B)**, Spearman correlation **(C)**, and X^2^ test **(L,M)**. TPM, transcripts per million. *P < 0.05, ***P < 0.001.

*KRAS, TP53, TTN, APOB*, and *COL6A1* were the most frequently observed mutated genes (Figure 2A). In accordance with previous studies showing a favorable prognosis in patients with a *MUC16-*derived neoantigen (2), we solely identified a *MUC16* mutation in the one patient still alive at the time of analysis (survival time of 157 months). A median of 54% [interquartile range (IQR) 12] and 30% (IQR 19) of all observed non-synonymous mutations resulted in MHCI-derived and MHCII-derived neoantigens, respectively, with 0.53–1.88 MHCI-binding and 0.38–42.02 MHCII-binding neoantigens produced per mutation. No difference was found between survival groups for quantity of non-synonymous mutations (*p* = 0.47; data not shown), MHCI-binding neoantigens (*p* = 0.47), or MHCII-binding neoantigens (*p* = 0.45; Figures 2B–D). In contrast to previous results, the presence of high CD8^+^ T cell infiltration together with neoantigen quantity was not associated with a more favorable prognosis (Figure 2E).

Next, we evaluated neoantigen quality in relation to survival. Quartile analysis of the similarity-to-self score indicated that tumor tissue from long-term survivors harbored less neoantigens in the least similar-to-self quartile (Q1) than short-term survivors (Figures 2F,G). This number of least similar to self-antigens in Q1 was correlated with plasma cell infiltration (r = −0.69; *p* = 0.01; Figure 2H), but not to CD8^+^ T cell infiltration or tumor stage (data not shown). INDEL-derived neoantigens (85% frame shift derived) were associated with this lower similarity-to-self score and short-term survival (Figures 2I–K). The type of INDEL (frame shift *vs*. deletion) was not associated with survival. Quartile analysis for the similarity-to-known-pathogen and immunogenicity score did not show differences between the survival groups (Figures 2L,M).

**Figure 3:**
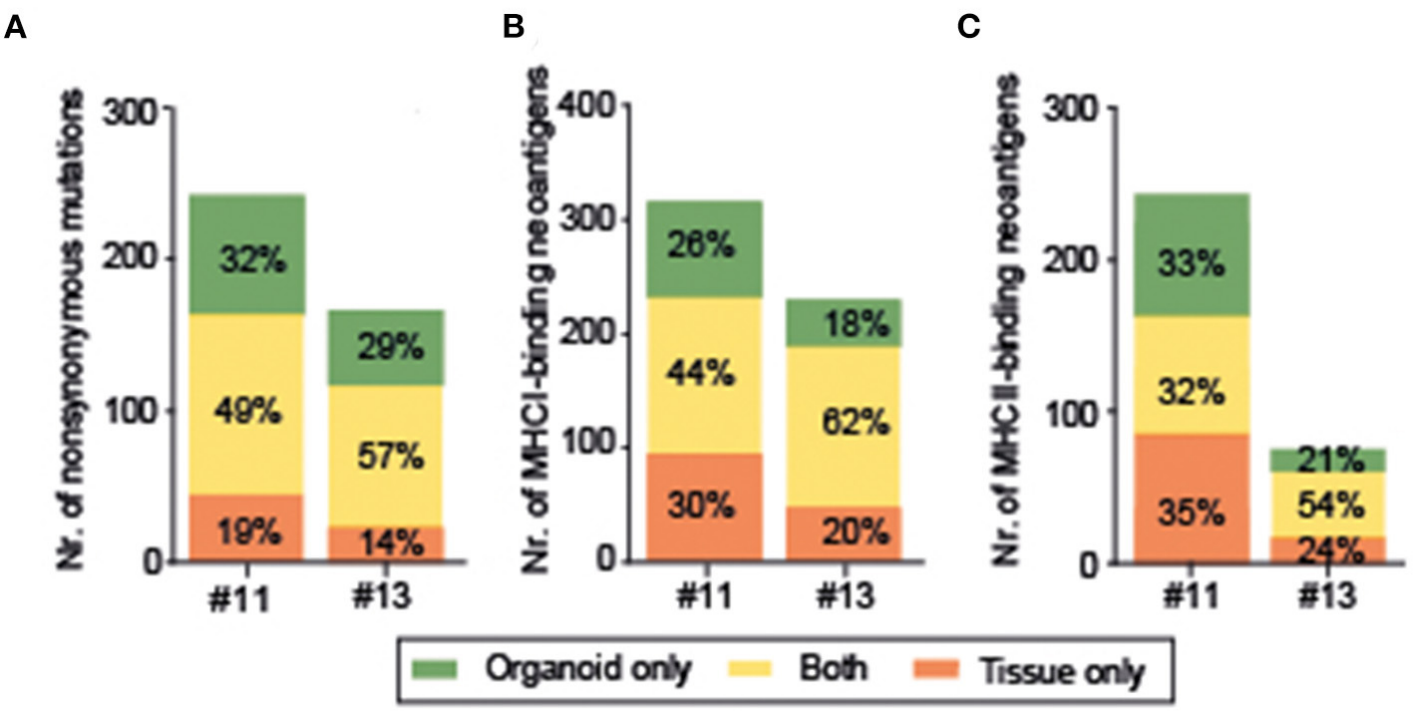
Organoids represent 49% and 57% of nonsynonymous mutations **(A)**, 44% and 62% of MHCI-binding neoantigens **(B)**, and 32 and 54% of MHCII-binding neoantigens **(C)**.

To evaluate whether organoids represent a good model for prediction of response to neoantigen-based immunotherapy, mutations and predicted MHCI- and MHCII-binding neoantigens were compared between original tumor tissues and corresponding organoids of two patients (#11, #13). Organoids and tissue matched regarding the presence of *KRAS, TP53, TTN, COL6A1* mutations, and the neoantigens derived from these prevalent mutations (Figure 2A). The percentage of INDELs was 11.4 and 9.0% in organoids, and 8.9 and 7.8% in tissue. For Patient #11, 390 MHCI-binding neoantigens were identified in organoid and 424 in tissue (Figure 3B); the median immunogenicity scores were 0.036 (IQR, 0.175) and 0.044 (0.227), self-similarity scores −6 (IQR 4) and −7 (IQR 3), and similarity-to-known-pathogen −19 (IQR 8) and −20 (IQR 8), respectively. For #13, 331 MHCI-binding neoantigens were identified in organoid and 368 in tissue; the median immunogenicity scores were 0.026 (IQR, 0.231) and 0.026 (0.252), self-similarity scores −4 (IQR, 4) and −6 (IQR, 4), and similarity-to-known-pathogen −18 (IQR, 8) and −18 (IQR, 8), respectively. About 49 and 57% of the non-synonymous mutations (Figure 3A), 44 and 62% of MHCI-binding neoantigens (Figure 3B), and 32 and 54% of MHCII-binding neoantigens (Figure 3C) were detected in both tissues and matching organoid lines (in thesame MHC-prediction context), respectively.

## Discussion

In agreement with previous results (2), our data confirm that neoantigen quantity alone is not associated with survival in patients with PC. We do, however, show that neoantigen quality is associated with survival time, even in this cohort without exceptionally long survivors. Counterintuitively, we demonstrate that shorter survival does not correspond with immunogenicity or similarity-to-known pathogen but rather with more neoantigens with a low similarity-to-self score, which appear to be derived from the higher number of INDELs or lower number of SNVs. While a high presence of INDELs is not related to increased survival in untreated patients, it is tempting to speculate that, as shown for other tumors (17), a higher presence of INDELs could be associated with a better response to immune checkpoint blockade in these patients. Thus, quality rather than quantity of neoantigens deserves more attention in PC research. In addition, further investigations into B-cell epitopes are required, as our data indicate that plasma cell infiltration may play a larger role in PC than expected (potentially independent of CD8^+^ T cell infiltration) (18). Future studies are needed that confirm these results in a larger sample size, and investigate the mechanism behind these findings.

For the two evaluated patients, organoids are able to recapitulate approximately half of the primary tumor-derived neoantigens and may be a suitable model for the prediction of survival. However, at first, it should be evaluated in a larger patient group. Additionally, as these organoids may have been grown from one or two clones present in the tumor, sequencing multiple organoids from each patient may increase this sensitivity. Furthermore, comparisons of cases and controls should be performed to evaluate if organoid culture induces new gene mutations, rendering it challenging to translate the data to neoantigen prediction for immunotherapy selection.

## Data Availability Statement

The data analyzed in this study is subject to the following licenses/restrictions: Another article will be generated. Requests to access these datasets should be directed to i.levink@erasmusmc.nl.

## Ethics Statement

The Erasmus and Maastricht Medical Center Ethical Review Boards approved the studies (MEC-2019-0135/METC 13-4-107). Both studies were carried out according to the ethical principles for medical research, involving human subjects from the World Medical Association Declaration of Helsinki.

## Author Contributions

IL contributed to study coordination, conceptualization, tissue collection, data curation, statistical analyses, and writing the original draft of the manuscript. LB carried out tissue and clinical data collection from 11 study participants, conceptualization, and writing of the manuscript (review and editing). SR performed the tissue and clinical data collection from two study participants, conceptualization, culturing of organoids, and writing of the manuscript (review and editing). MA contributed to tissue and clinical data collection from two study participants, conceptualization, culturing of organoids, and writing of the manuscript (review and editing). SOD performed tissue and clinical data collection from two study participants, conceptualization, culturing of organoids, and writing of the manuscript (review and editing). DC contributed to conceptualization, supervision (daily), and writing of the manuscript (original draft, review, and editing). SB performed conceptualization, transcriptomic analysis (CIBERSORTx), supervision, and writing of the manuscript (review and editing). GF participated in conceptualization, supervision (daily), and writing of the manuscript (original draft, review, and editing). MP is the head of the laboratory; he created an infrastructure that enabled performing the study, contributed to conceptualization, supervision, and writing of the manuscript (original draft, review, and editing). MB is the principal investigator, who created the infrastructure that enabled performing the study, performed conceptualization, supervisor, and writing of the manuscript (original draft, review, and editing). All authors contributed to the article and approved the submitted version.

## Conflict of Interest

SR: A shareholder of Adjutec B.V. SOD: A shareholder of Adjutec B.V. MB: Boston Scientific (Consultant, support for industry and investigator-initiated studies), Cook Medical (Consultant, support for industry and investigator-initiated studies), Pentax Medical (Consultant, support for investigator-initiated studies), Mylan (Support for investigator-initiated studies), ChiRoStim (Support for investigator-initiated studies). LB served as a paid consultant for Bristol-Myers Squibb in Pathologist Advisory Board PD-L1 CPS testing in upper GI cancer. The remaining authors declare that the research was conducted in the absence of any commercial or financial relationships that could be construed as a potential conflict of interest.

## Publisher's Note

All claims expressed in this article are solely those of the authors and do not necessarily represent those of their affiliated organizations, or those of the publisher, the editors and the reviewers. Any product that may be evaluated in this article, or claim that may be made by its manufacturer, is not guaranteed or endorsed by the publisher.
